# Barriers and opportunities to bridge between hospital and community via rehabilitation exercises for people with disabilities: multi-ministerial R&D efforts in South Korea

**DOI:** 10.3389/fresc.2025.1505943

**Published:** 2025-06-04

**Authors:** Hogene Kim, Aerim Kim, Hye Min Choi, Jungah Lee, Jung Hwan Kim, Hyosun Kweon

**Affiliations:** National Rehabilitation Center, Ministry of Health & Welfare, Seoul, Republic of Korea

**Keywords:** rehabilitation exercise, R&D, disability, smart exercise equipment, data continuity, health promotion

## Abstract

People with disabilities often experience limited participation in community-based exercise activities aimed at promoting health. The concept that “Exercise is Medicine” is widely acknowledged across societies and historical periods. However, there is a notable discontinuity between hospital-based and community-based health promotion efforts for people with disabilities. This article discusses multi-ministerial research and development (R&D) efforts in South Korea to address this issue, emphasizing the need for transitional strategies to successfully bridge the service gap. As the first collaborative multi-ministerial R&D initiative between the sports and clinical ministries in South Korea, this project focuses on community-based rehabilitation exercises through three major components: (1) Smart Exercise Equipment, (2) Disability-based Exercise Programs and Services, and (3) Data Continuity on Health Information. A standardized community rehabilitation exercise dataset was also developed to evaluate activities of daily living, primarily categorized into physiological outcomes during exercises, clinical assessments, and lifelog data measurements. The National Rehabilitation Center, under the Ministry of Health & Welfare and serving as the leading ministry, is dedicated to developing a rehabilitation exercise Living Lab that integrates these three components in collaboration with other ministries. This initiative aims to benefit people with disabilities by enhancing their health through data-driven rehabilitation exercise services. Furthermore, clinicians and community rehabilitation exercise providers could improve services by utilizing this standardized dataset, facilitating comparisons of clinical records through a public service platform.

## Introduction

People with disabilities (PwDs) often face limitations when participating in community-based activities (1–3). Simple exercise programs that are accessible to PwDs can become a valuable tool in promoting their health and functioning (4, 5). These types of community exercises would include passive-type exercises on balance, strength, aerobic activity, and community sports, e.g., basketball, pin ball, tennis (6, 7).

Well-established infrastructure for indoor and outdoor exercise programs and activities can significantly promote health in PwDs (8, 9). Regardless of disabilities or diseases, numerous ways have been implemented to promote health in older PwDs, e.g., the multifactorial fall-prevention program (10), cancer survivor support programs (11), and cardiorespiratory fitness programs for stroke survivors (12). There has also been a growing interest and commitment from government ministries and local authorities to promote community exercise initiatives, in South Korea, as evidenced by the 2022 National Status Reports on Public Sports Facilities reported by the Ministry of Culture, Sports, and Tourism (MoCST) (13).

“Exercise is Medicine” is a well-known belief in virtually every society and historical periods (14–18). Early evidence of this recognition can be found in writings of Hippocrates who prescribed gait exercise as a treatment for consumption as early as 500 B.C (19). In promoting health by connecting exercise and clinics, Rehabilitation Medicine has played a central role in highlighting this concept. This was evidenced in the implementation of exercises and public health programs in 2007 and following guidelines by the American College of Sports Medicine (ACSM), American Medical Association, American Heart Association, and the Office of the Surgeon General, as initiatives to promote exercise, prevent disability, manage diseases, and improve quality of life (20–23).

The spectrum of disabilities is diverse. Under the Act on Welfare of Persons of Disabilities (24) or International Classification of Functioning, Disability and Health (ICF), two main groups exist—physical and mental disabilities. Under the first main group of physical disabilities in Korea, there are six subgroups of external function disabilities: physical, brain injury (e.g., stroke), visual, auditory, language, and facial. Further subdivisions in the physical disability subgroup include amputee, musculoskeletal (e.g., joint disorders), and neurological (e.g., spinal cord injury) disabilities. There are also six subgroups of internal function disabilities under the first main group: kidney failure, cardiac disorders, liver malfunction, respiratory disorders, toileting difficulties, and epilepsy. Under the second main group of mental disabilities, we have developmental disabilities (e.g., autism) and mental disorders (e.g., schizophrenia, depression, anxiety). Notably, the first five physical disability subgroups comprise 79.9% of the 2.5 million PwDs in South Korea (25). Therefore, healthcare programs addressing these different kinds of disabilities should be developed to provide the necessary management based on the patient's disability.

**Figure 1:**
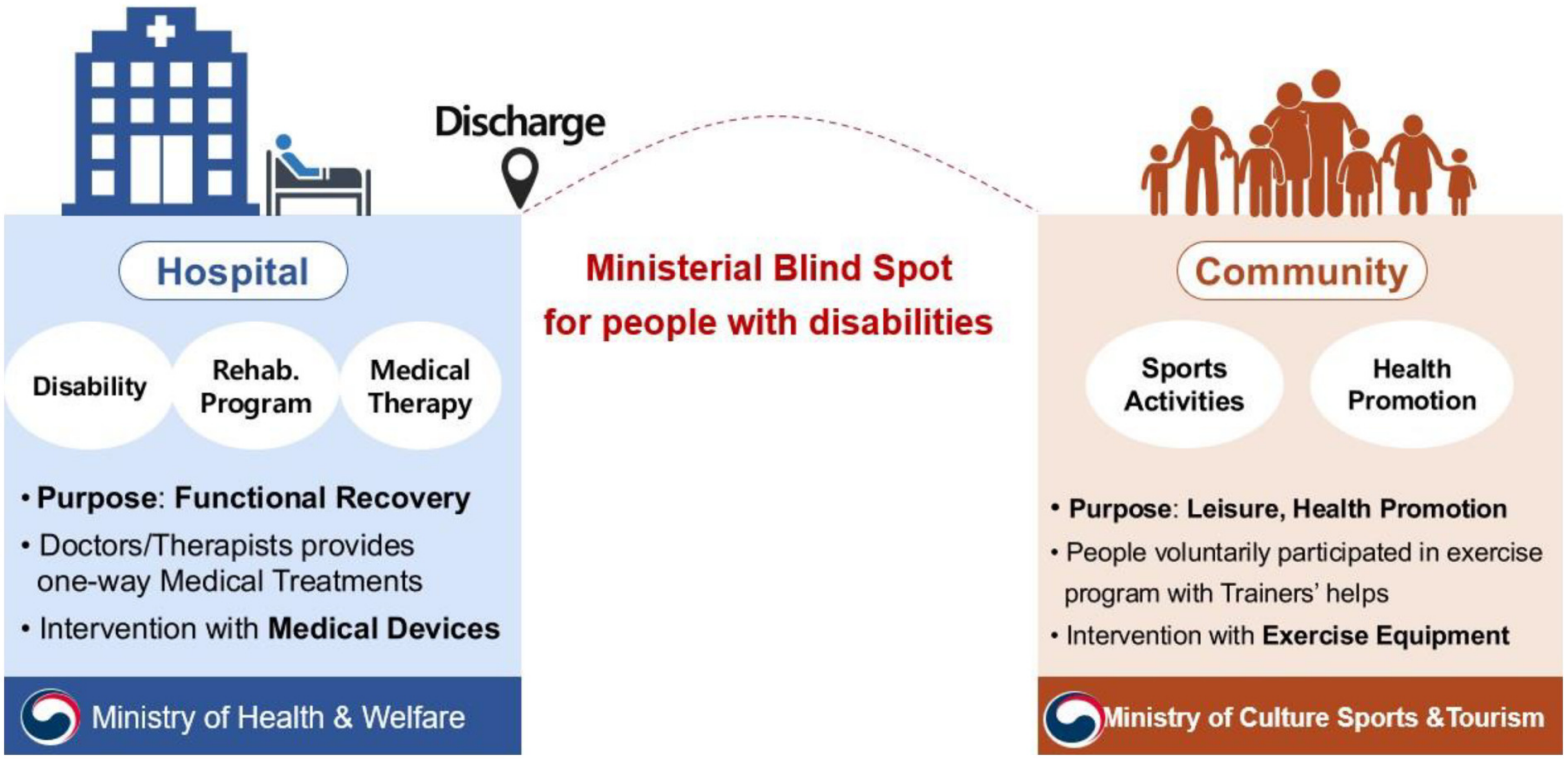
Ministerial blind spot for health promotion in people with disabilities.

Figure 1 illustrates the blind spot between the hospital and community settings for health promotion in PwDs. Within hospitals, licensed medical doctors and therapists are the only authorized individuals to provide functional recovery services, which are mostly one-way, medical treatments. The provision of such services by non-licensed clinicians is strictly prohibited by law. Furthermore, the Ministry of Health and Welfare (MoHW) enforces administrative orders aimed at promoting the health of PwDs under the medical law. In contrast, the community provides voluntary activities focused on sports and exercises for the purpose of leisure and health promotion. However, the National Sports Promotion Act mandates that only certified physical trainers can provide special training services to PwDs, especially those with more stable conditions. Moreover, certified physical trainers cannot provide any medical treatments in the community, while clinicians are prohibited from providing treatments for PwDs outside the hospital. This inevitably creates a blind spot, particularly regarding health promotion for PwDs. Due to the ambiguity in administrations and conflicting legal frameworks, this blind spot between the MoHW and MoSCT remains unaddressed in any research and development (R&D) efforts. Consequently, discharged patients with unfamiliar disabilities would often struggle to find continuity in their health services. This lack of support can lead to feelings of isolation, depression, and associated symptoms. Although attempts have been made to address this issue by both sectors, significant gaps remain. For instance, the MoHW implemented the Community-based Rehabilitation (CBR) program which provided medical rehabilitation services in the community, and the MoCST established community-based exercise services for PwDs. However, both programs have yet to bridge the disconnect between clinical and community-based rehabilitation services.

Another significant factor contributing to this disconnect is the administrative separation between the medical and community health system, which is common in most developed countries. In South Korea, the MoHW oversees the medical system, whereas the MoCST governs the community health system (26). A recent legislative development, the “Act of Health Promotion and Access to Medical Services for Persons With Disabilities (Act of H&APWD),” reflects the culmination of long-standing efforts by disability groups to advocate for the right to community-based health programs. Despite this, the title of the 15th statement, which is “(Therapeutic) Rehabilitation Exercise and (Community) Rehabilitation Training,” suggests lingering disagreements between the medical and sports worlds regarding the scope of rehabilitation services. This is especially true since PwDs would be more inclined government assistance, such as disability vouchers and health insurance (e.g., Medicare, Medicad).

Aside from administrative issues, studies showed that a major hurdle for community-based rehabilitation (CBR), especially for PwDs, is due to the lack of knowledge and equipment (27, 28). Hospital-grade exercise equipment is typically unavailable or unaffordable for those in the community. This creates discrepancies that lead to inconsistencies and discontinuity of exercise routines. For instance, a stroke survivor intending to continue their rehabilitation exercises at home may struggle to find appropriate equipment that is both safe and effective, especially compared to those receiving specialized rehabilitation exercises (REs) in a hospital setting. This is a similar issue in rehabilitation programs tailored to specific conditions but lack the necessary resources in the community. Clinically proven services and meticulous observations are crucial for the safety and effectiveness of REs in the hospital setting to be properly translated to a community setting. Doing so would ensure continuity of care and optimization of medical costs. A collaboration between both settings through seamless communication would be essential for this to happen.

Given all these, this article describes governmental R&D efforts in South Korea to help resolve the current issue in health promotion, highlighting the need for constant communication and transition efforts for the successful bridging of services from the hospital to the community.

### R&D efforts

Previous studies have reported a persistent distrust between the field of sports and medicine regarding RE, as both fields view RE as exclusively within their domain. This contributes a broader lack of coordination and collaboration in rehabilitation efforts. In the Inter-Ministerial Competition on R&D project planning strategy in 2020, our proposal won first place after three rounds of evaluations. Although 108 teams applied for the 1st round, 36 teams advanced after the 2nd round, and only 12 teams were presented in final round. Our final presentation garnered the support of 10 deputy directors from participating ministries, ultimately securing a well-deserved first place and a grant of 50 million dollars for the next 3 years.

Our current initiative represents the first collaborative R&D project between the MoHW and MoCST that focuses on community-based REs for PwDs. However, despite offering similar services in the same target population, these two sectors differ significantly in their approach. Whereas the hospital-based approach relies on therapists and physiatrists in specialized institutions, the community-based approach makes use of physical trainers in gyms. This disparity can create challenges when navigating the gray area between the two sectors, such as the sensitive terminology use of “sports” and “clinical.” Even the use of the term “RE” was insisted by both sectors to be their own term.

To address this, we strategically sought a collaborative platform between the two sectors. This led to the successful involvement of the Public R&D Policy Division of the ministry of Science & ICT (MoSICT) and subsequent participation of the Director of National Public Research Strategy, who was the associate head of the National Science Foundation in South Korea. Following the second round of evaluations, the project's initial ideas were shared across various ministries, which garnered the participation of the Administration of Forest (AF). AF saw a clear alignment with our initiative, as they had already offered similar community services on health promotion for PwDs at the national or public forests. In fact, the insights of the AF program, such as group meditation sessions in the forests, were positively received by PwD participants. This multi-ministerial collaboration culminated in the development of a novel R&D program in South Korea.

### R&D program components

Our R&D programs consist of three major components: exercise equipment and sensors, programs and services, and data continuity. To ensure that all these components are fulfilled, a living lab in the National Rehabilitation Center (NRC) works to assess if these programs would bridge REs for PwDs between the hospital and community settings.

1. Smart Exercise Equipment

Various rehabilitation equipment was developed, categorized as either 1st or 2nd class medical devices, to target the upper and lower extremities, as well as the whole body. Complementing this equipment are advanced wearable and portable sensors, such as Inertial Measurement Units or Image Sensors. These sensors are designed to track REs and quantify the amount of exercise performed, following the FITT principles (Frequency, Intensity, Time, Type) specific to each individual's disability (21).

2. Disability-based Exercise Programs and Services

Disability-specific RE programs were initiated based on the clinical and community settings. Thus, with regards to requests for proposals (RFPs), applicants would either become disability trainers in the communities or rehabilitation clinicians in the hospitals.

3. Data Continuity on Health Information

To ensure continuity of rehabilitation services from the hospital to the community, multi-ministerial efforts in R&D have been undertaken. One example is the “Public MyData” project in South Korea, which involves government intervention in personal data management within the finance and healthcare sectors. This project allows individuals to consent to the use of their personal data for public service benefits, such as personalized healthcare, with enhanced security using data encryption. Moreover, the data is aggregated into a fractionalized big data pool, which can be utilized for further improvements in health services including PwDs.

Under South Korean law, individuals have the right to request physical or digital copies of their medical records, including therapies and evaluations, upon hospital discharge. During hospitalization, PwDs are likely to have undergone numerous assessments and evaluations during rehabilitation sessions, such as the Berg Balance Scale, Fugl-Meyer Assessments, Joint Range of Motion testing, and Manual Muscle testing.

**Figure 2:**
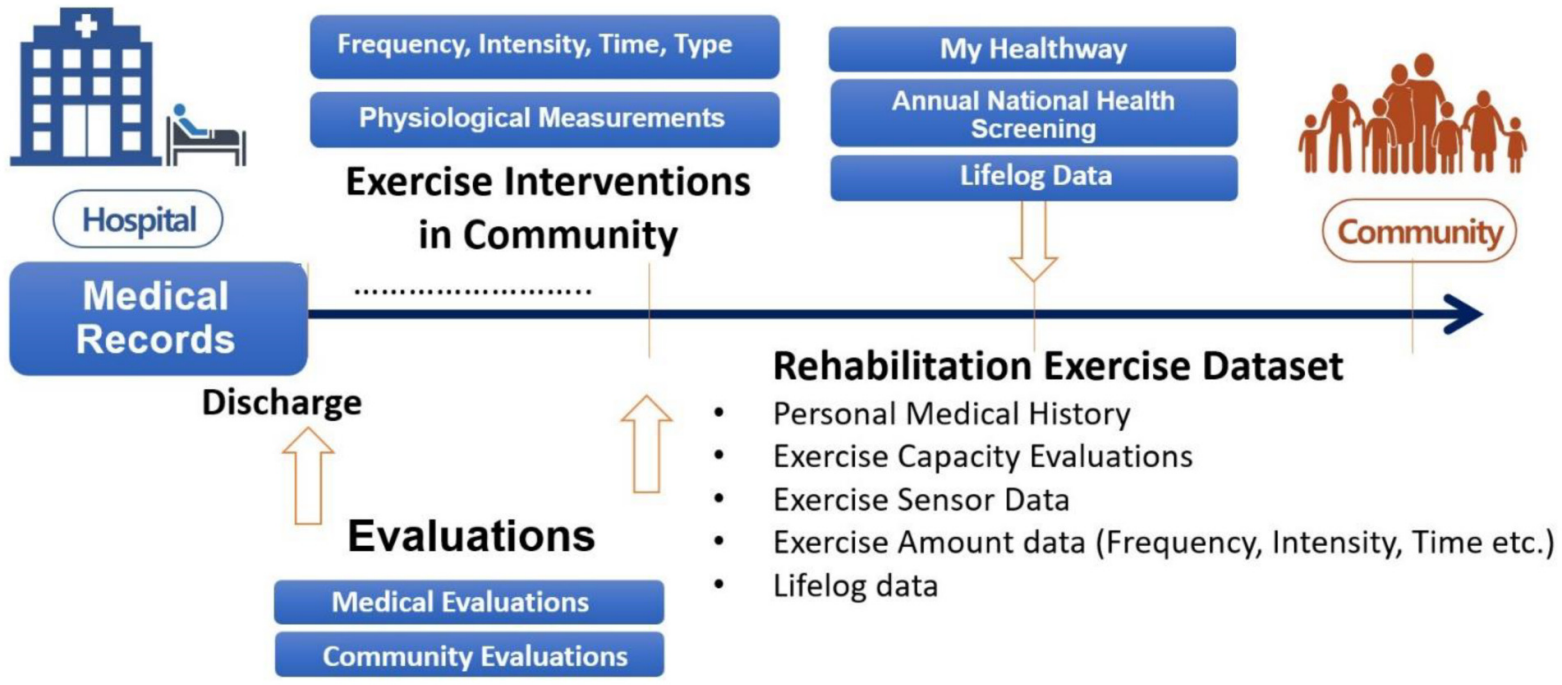
Conceptual model to provide continuous rehabilitation service via rehabilitation exercises to people with disabilities.

Despite the availability of medical evaluation data, patients often lack the means to utilize them effectively, especially since medical rehabilitation records become inaccessible and unusable after hospital discharge. This marks the beginning of the gap in rehabilitation services for PwDs. To resolve this, we used the MyData platform to record patients' exercise frequency, intensity, time, and type within the community. As illustrated in Figure 2, this data, coupled with clinical or community evaluations recorded at regular intervals, will enable clinicians and exercise specialists to provide personalized healthcare treatments that can be refined based on gathered data. The RE dataset consists of five key categories to improve healthcare services: Personal Medical Records, Exercise Capacity Evaluations, Exercise Sensor Data, Exercise Amount Data (FITT), and lifelog data. Using this dataset, clinicians can access standardized data forms containing both clinical-based and community-based evaluations during check-ups. Physiological measurements can also be safely stored and closely monitored in the MyData platform. Additionally, in South Korea, the MyData platform can be linked with the Annual National Health Screening data and lifelog data in the MyHealthway project to further enhance health promotion in the community setting.

### Initial proposal of RE dataset definitions

A refined definition of the community RE dataset (available upon request) is needed to evaluate activities of daily living among PwDs. Considering its five key categories, the initial proposal underwent focus group interviews and consultations with experts in the field, including physical medicine & rehabilitation doctors, physiatrists, national paralympic trainers, community physical trainers, and scholars from various fields. The specific process of the proposal is described in the following types of information.

First, participants consent to the storage of their basic personal information and medical records in the MyData platform, which is a public network of data. Second, initial RE dataset is arranged into two major categories: hospital-based and community-based evaluations. Both approaches share the same fundamental measurements for RE—basic joint movement and muscle strength [e.g., passive and active joint range of motion (ROM), manual muscle testing (MMT)]. Although maximal MMT and joint ROM were initially proposed as sole fundamental measurements to one of the approaches, there were concerns regarding variability and the need for additional input values. Thus, it was reasonable to include these measurements for both settings, acknowledging differences in their application. In the hospital setting, comprehensive clinical tests are performed to assess various functions, including Fugle-Meyer Assessment (FMA), Brunnstrom Staging, and Berg Balance Scale (BBS) scoring, whereas in the community setting, simpler function tests are conducted, including ROM, MMT, and the Short Physical Performance Battery (SPPB). Third, physiological sensor measurements are measured with modern exercise equipment. Conventional physiological outcomes, which are evaluated during exercise, include equipment-specific information, such as speed and distance for treadmills and bikes, heart rate, and oxygen saturation levels (SpO_2_). Fourth, as the most crucial data in the system, the FITT of REs is evaluated. To comprehensively track and monitor REs in the community, a well-defined dataset that includes hospital data (e.g., health screening tests, prescriptions, blood tests) is essential. Various clinical assessments for the evaluation of different functions can be done by certified professionals in the clinical and community settings. Comprehensive functional tests generally done by certified clinicians include FMA, Timed Up and Go test (TUG), 10-Minute Walking Test (10mWT), Functional Ambulation Category (FAC), BBS, and Brunnstrom Staging. Psychological tests [MOCA-K, Depression Test, Pain Scale, MMSE (Mini-Mental Status Examination)] and social function tests (EQ-5D-3l) are also included due to their significant influence on rehabilitation outcomes. It should also be noted that some of these measurements (e.g., FMA, BBS, MOCA-K) are often done in South Korean clinics due to their coverage under national health insurance. Lastly, the emergence of wearable sensors opens up the possibility of recording lifelog measurements, which are measured from smart wearable devices (e.g., Apple watch, Fitbit, Galaxy Fit). This data, encompassing exercise records, sleep patterns, and pedometer readings, can be compared to clinical assessments and physiological outcomes by trainers and clinicians.

### Data collaborations—information flow model

**Figure 3:**
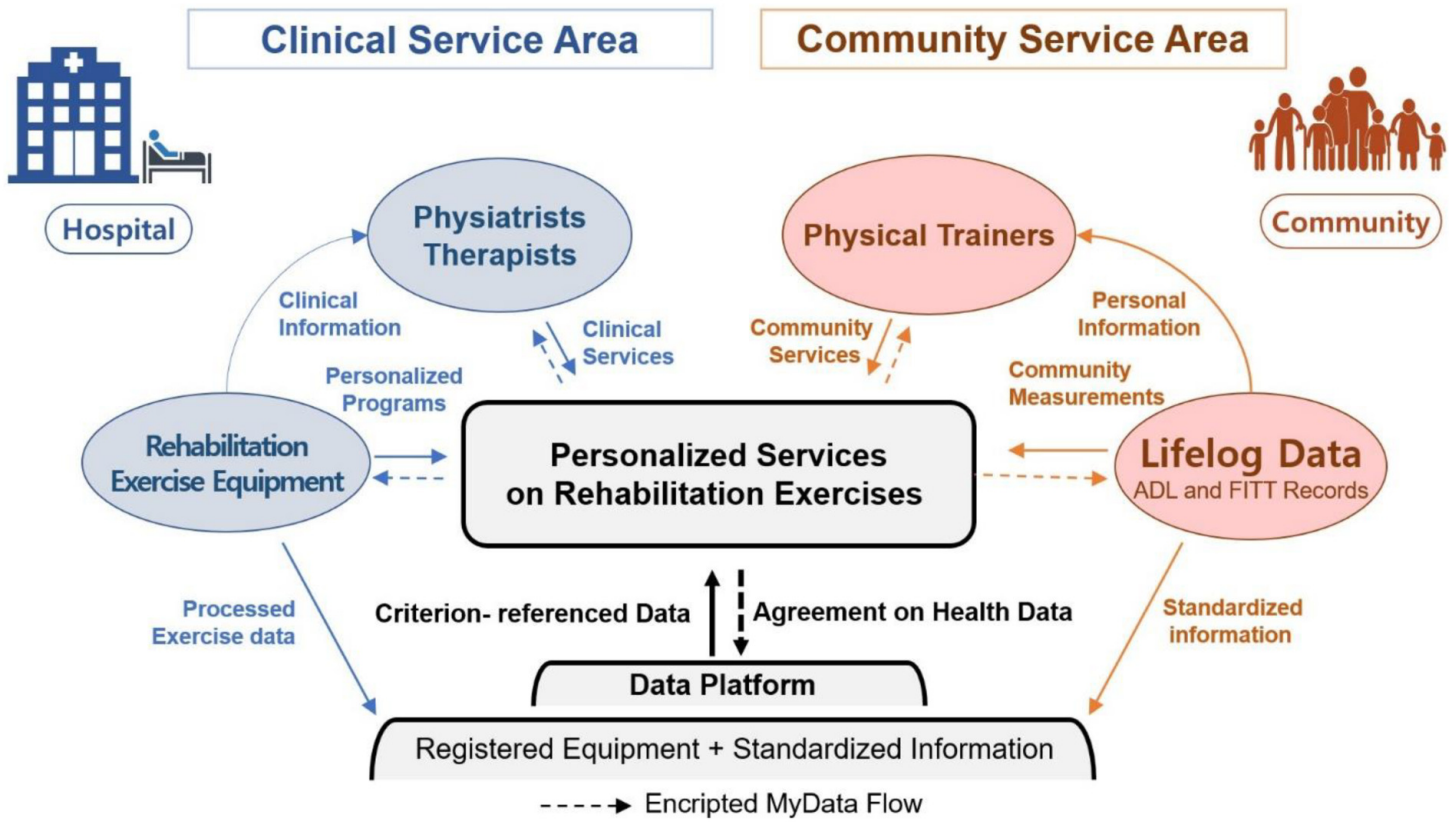
Data collaboration model to provide personalized rehabilitation exercise services to people with disabilities.

Figure 3 illustrates the MyData flow model for the provision of personalized RE services to PwDs. The purple figures represent the recipients of these services, the blue figures represent the hospital, and the orange figures represent the community. The MyData platform aids in healthcare services of both the hospital and community settings. Notably, MyData-based services were also developed as means to promote preventive eHealth services (29).

In the hospital setting, participants who agree to share their medical records in the secure MyData platform can receive personalized RE services from licensed clinicians. This model leverages medical exercise equipment to record and store personalized RE data in the MyData platform (29, 30). Rehabilitation clinicians are then able to provide evidence-based medical treatments using hospital-grade equipment, and RE records are subsequently standardized (e.g., FITT, sensor measurements) and transferred to the platform. An example of this process has been observed with the use of electronic medical records (EMRs) to take note of exercise duration and type (e.g., cardiac exercise).

In the community setting, RE and lifelog data equipment keep track of the mobility and physiological records of PwDs during community-based exercise programs. Data are then recorded in the public MyData-based platform, which is securely encrypted as per the patients' agreement.

### NRC rehabilitation exercise living Lab

**Figure 4:**
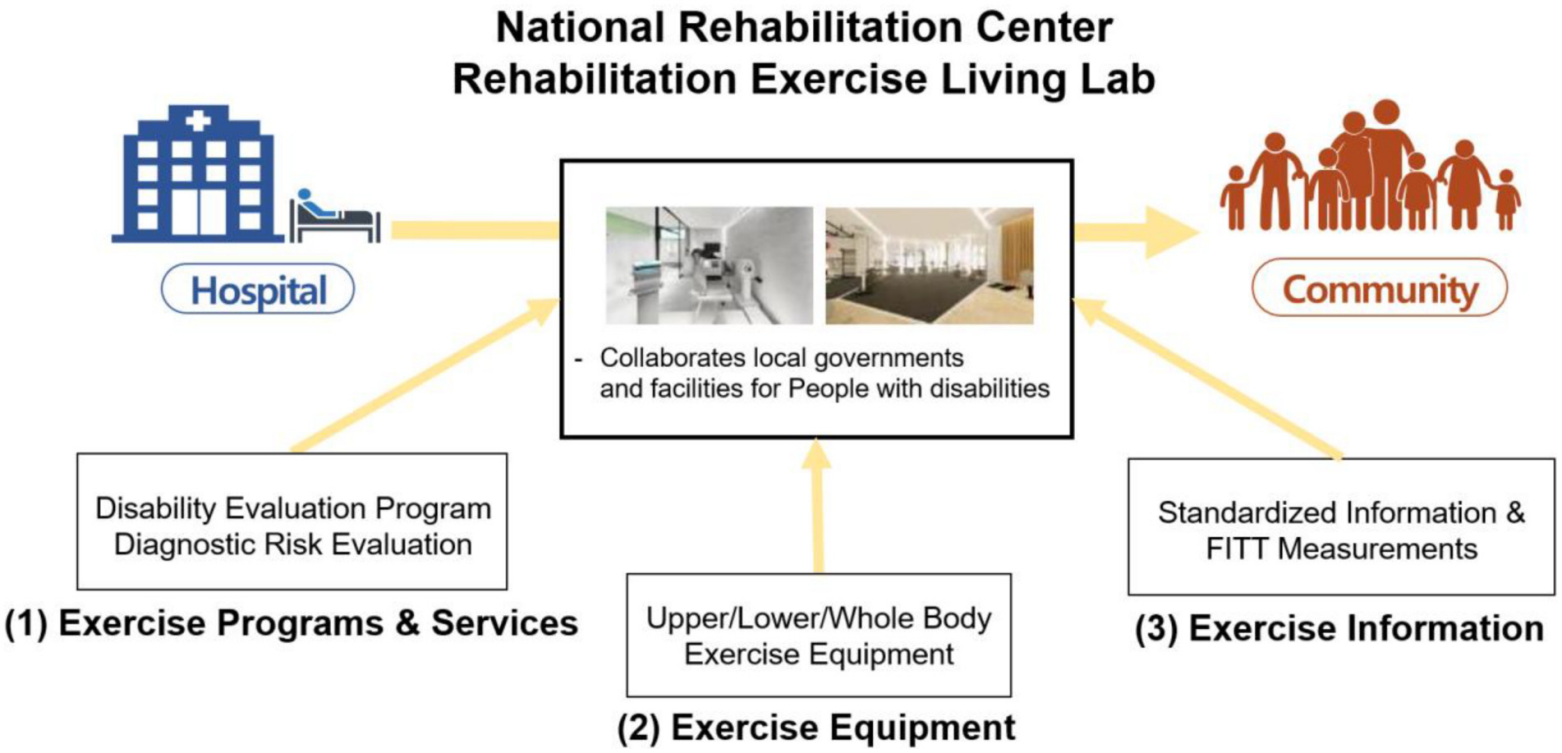
Rehabilitation exercise living Lab in national rehabilitation center.

The NRC stands as an ideal living lab for testing and implementing the proposed project. This facility combines the resources of the largest national rehabilitation hospital in South Korea with experienced rehabilitation clinicians and specialized exercise trainers, fostering an environment where gaps between community and hospital REs can be bridged. The NRC focuses on three development areas: Programs and Services, Exercise Equipment, and Data Services. As illustrated in Figure 4, individuals would transition through the living lab upon discharge from the hospital. Doing so will allow PwDs to learn about and experience the rehabilitation exercise programs and services using proper equipment tailored to their disability. Data gathered during this period are then recorded and stored within the community data platform, providing medical records for further management and valuable insights for optimizing rehabilitation plans.

### Multi-ministerial R&D program—collaborations and structure

**Figure 5:**
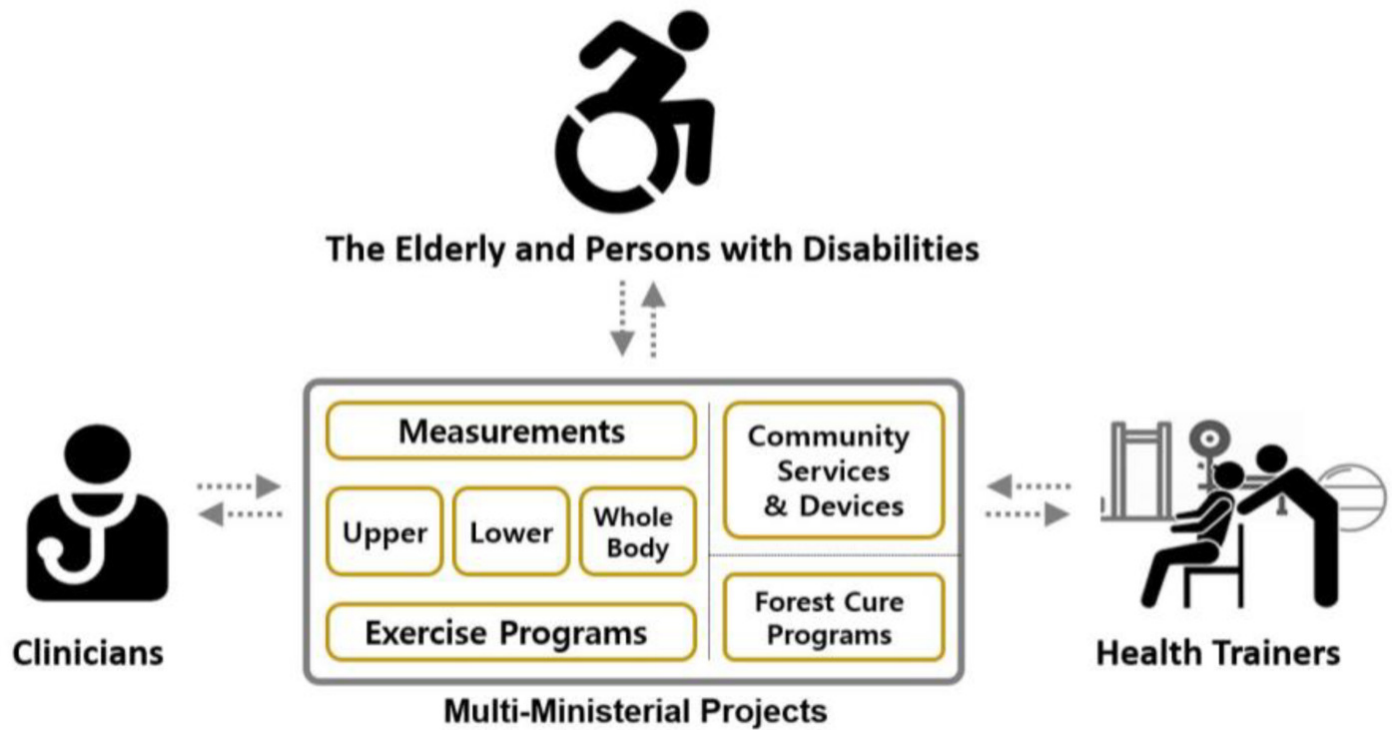
Multi-ministerial R&D projects.

This project marked a significant milestone, being the first initiative to bring together stakeholders from different ministries who participated in multi-ministerial R&D projects (Figure 5). To ensure seamless collaboration and neutrality, numerous multi-ministerial meetings were held under the supervision of the Public Division Chair of the Korean National Science Foundation. The MoHW acted as the leading ministry, in collaboration with the MoCSR, MoSICT, and AF. Participants included deputy directors, who oversee policies and budgets, and administration agency officials, who manage funding and evaluation, from each ministry. Research representatives from the three participating ministries were directly involved in biannual grand meetings, where they interacted with other stakeholders, including PwDs, rehabilitation clinicians, and medical/commercial/community specialists. The interests and concerns of these stakeholders are diligently addressed, prompting ministerial agencies to make adjustments to the project's direction through the use of RFPs under the unanimous supervision of the deputy directors. With the MoHW at the helm of this multi-ministerial project, what once was a concept had become a successful and effective endeavor in providing appropriate healthcare in the whole community.

### Barriers of collaboration—three different languages

The greatest barrier to successful collaboration lies in bridging the gaps between diverse expertise areas. A data-driven, public service that is accessible and responsive can have an immediate impact on the service market. However, clinical trials and evidence-based medicine adhere to strict procedural protocols (e.g., clinical regulations) to reach a widely accepted conclusion. In medical informatics, this involves defining new medical flow charts for treating diseases using innovative technologies, which is a complex process.

### Opportunities in rehabilitation exercises

Leveraging the data accumulated through progressive measurements of REs presents numerous opportunities in improving the lives of PwDs. This includes personalized treatment plans, regular clinic visits, and comprehensive training services. In addition, nationwide benefits for PwDs extend to cost reductions in healthcare services. Following the successful definition of core measurement parameters, ongoing projects across participating ministries have actively shared datasets for the improvement of health promotion among PwDs.

This initiative benefits both older persons and PwDs through the promotion of their health and provision of services from the hospital and community settings. Clinicians may also benefit from this project as an opportunity to develop better clinical services with reference to standardized community data and community rehabilitation exercise datasets, allowing comparisons of clinical records through the service platform. This access to data also empowers health training specialists to provide more reliable training by minimizing injury risk for PwDs.

## Data Availability

The original contributions presented in the study are included in the article/Supplementary Material, further inquiries can be directed to the corresponding author.
